# Observations Respecting Inoculation

**Published:** 1805-02-01

**Authors:** W. King


### Observations respecting Inoculation; *by* W. King, M. D.

1. Having inoculated with cow-pox matter, some children who had been inoculated with small-pox three years before, a very slight local irritation only could be produced.

2. On trying the variolous matter, after the vaccine, no effect whatever was produced.

3. I made use of vaccine matter taken from the arm so early as the eighth, and so late as the sixteenth day from inoculation, with equally complete success.

4. Having inoculated a number of children with matter, (vaccine) which had been kept between two pieces of glass for two months, only one in nine missed infection.

5. In one instance I inoculated with vaccine matter, with equal care, *three times* before it took effect.

6. I inoculated (with cow-pox matter) a lady near sixty years of age, who went through the different stages of the disease! She had brought up a family of ten children, and attended them all in small-pox by the natural contagion, without ever having had the disease herself; but was always ways under great apprehensions of it. Two of her children were inoculated with the vaccine matter at the same time with herself; in them no effect was produced, while she had this disease favourably, and thus was freed both from the danger and dread of the other.

7. An instance of cow-pox, measles, and hooping cough, in the same person, at the same time, as under. I was called to visit a maid-servant ill of the measles, at a village where a number of children had the hooping cough; she had been there only a few days, and had come from another village, where measles had lately prevailed. I had proposed to inoculate all the children of this village with cow-pox gratis; but the measles having appeared, the lady of the house wished to know if it would be proper to do so at the time? I replied, that as to the cow-pox as a disease, no danger whatever was to be apprehended from it; and as the hooping cough prevailed, there was great reason to believe that it would much alleviate, if not altogether relieve some from it; besides, I thought there was a probability that it would prevent the contagion of measles from spreading. The lady did me the honour of assenting to these ideas, and thought the people would all be glad to avail themselves of so kind and generous an offer. Accordingly they all, to a few, brought their children to be inoculated.

In the house nearest to the hall were three children; one of them four years old, another two, the third a few months; the oldest child, a boy, was occasionally in the kitchen where the servant was, under measles; about the usual time his arm inflamed, and also the measles appeared; his cough became much easier; all symptoms pretty mild.

The youngest child not having taken the cow-pox, I repeated the inoculation in the one arm from the pustule of the child with measles, and in the other from that of the second child who had not measles.

The infection took place in both arms, and I could perceive no difference in the appearance of the pustules, nor did the infant shew any sign of measles; its cough, however, was also relieved during the vaccination; and what was observable, the measles seemed so to have protracted, or retarded the progress of cow-pox in the oldest child, that the arm of the young one, inoculated from it, was healed up before his.

No other child in the village took measles, which I apprehend might be attributed to vaccination; all of them were, more or less, relieved of hooping cough during it.

Another

Another servant girl, who came into the house afterwards, took the measles; yet, even after that, they did not spread in the village.

Fully convinced of the great value of the Jennerian discovery, I had endeavoured to extend its utility to the poor; and with this view, at one village, I offered to the Farmer to inoculate all the children on his extensive farm without any expence. He sent his servant to inform them, and lest any mistake had been committed, he went himself, and returned with the same report as he had received, that none of the parents would tempt or offend the Lord by bringing disease on their children, but would trust to Providence! He thanked me kindly for so generous an offer, but was very sorry his dependents would not avail themselves of such liberality, of which, from their conduct and ignorant superstition, they proved themselves unworthy.

*Respecting Scarlatina Anginosa.*

Chiefly in the months of September and October last, prevailed the scarlatina anginosa, which much resembled that described by Dr. Sims, as met with in London in 1786. Several anomalous cases were met with, some few died. In some the eruption was considerable, the throat little affected, and the contrary. Sometimes the throat appeared ulcerated very early, and in others late in the disease, and in several the sebaceous crust only appeared. In some the inflammatory symptoms were brisk at first, in others they had a putrid tendency.

Going one day to visit a family ill of it, one had the eruption very complete, with strong fever from the first; it occured to me, without being conscious of having ever heard of any similar suggestion, that it might be communicated by inoculation. I prevailed on the parents to submit one child to it. The result was, that the arm inflamed, and the child took the disease much as in cow-pox, and the febrile symptoms were so slight that the child was not at all confined thereby; the vesicles filled with laudable pus, and gradually dried off. I afterwards inoculated the child with cow-pox, and it took the disease very favourably.

Another child, very like it in age, constitution, &c. was inoculated with cow-pox, and its arm was the most inflamed and ulcerated of any I met with during the year.

*Query.* Might not the Scarlatina, by inoculation, have caused the other to be more easy?

May not Scarlatina be safely inoculated for?

May it prevent Small-pox, or other Exanthemata?

